# Phenol-Soluble Modulins Modulate Persister Cell Formation in *Staphylococcus aureus*

**DOI:** 10.3389/fmicb.2020.573253

**Published:** 2020-11-09

**Authors:** Mara Baldry, Martin S. Bojer, Zahra Najarzadeh, Martin Vestergaard, Rikke Louise Meyer, Daniel Erik Otzen, Hanne Ingmer

**Affiliations:** ^1^ Department of Veterinary and Animal Sciences, Faculty of Health and Medical Sciences, University of Copenhagen, Frederiksberg, Denmark; ^2^ Interdisciplinary Nanoscience Center (iNANO), Aarhus University, Aarhus, Denmark

**Keywords:** phenol-soluble modulins, persister cells, biofilm, fibrils, accessory gene regulator, *Staphylococcus aureus*, PSM, agr

## Abstract

*Staphylococcus aureus* is a human pathogen that can cause chronic and recurrent infections and is recalcitrant to antibiotic chemotherapy. This trait is partly attributed to its ability to form persister cells, which are subpopulations of cells that are tolerant to lethal concentrations of antibiotics. Recently, we showed that the phenol-soluble modulins (PSMs) expressed by *S. aureus* reduce persister cell formation. PSMs are a versatile group of toxins that, in addition to toxicity, form amyloid-like fibrils thought to support biofilm structures. Here, we examined individual or combined synthetic PSMα peptides and their equivalent amyloid-like fibrils on ciprofloxacin-selected *S. aureus* persister cells. We found that PSMα2 and the mixture of all four alpha peptides consistently were able to reduce persister frequency in all growth phases, and this activity was specifically linked to the presence of the soluble peptide as no effect was seen with fibrillated peptides. Persister reduction was particularly striking in a mutant that, due to mutations in the Krebs cycle, has enhanced ability to form persisters with PSMα4 and the combination of peptides being most effective. In biofilms, only the combination of peptides displayed persister reducing activity. Collectively, we report the individual contributions of PSMα peptides to persister cell reduction and that the combination of peptides generally was most effective. Strikingly, the fibrillated peptides lost activity and thus, if formed in bacterial cultures, they will be inactive against persister cells. Further studies will be needed to address the biological role of phenol-soluble modulins in reducing persister cells.

## Introduction

*Staphylococcus aureus* is notoriously famous for being an opportunistic pathogen and causative agent of a wide array of diseases in humans and animals alike (Lowy, 1998). The disease-causing abilities lay partly in the plethora of virulence factors that *S. aureus* can produce (e.g., adhesins, proteases and toxins) and the ability of the organism to form biofilm (Lowy, 1998; IWG-SCC, 2009; Archer et al., 2011). Biofilm is an organized multicellular bacterial community embedded in a matrix composed of proteins, polysaccharides, and extracellular DNA. Grown in biofilms *S. aureus* often results in persistent and chronic relapsing infections due to being particularly recalcitrant to antimicrobials and host defenses, thus contributing substantially to the morbidity and mortality associated worldwide with *S. aureus* infections (Lowy, 1998; Archer et al., 2011).

Both biofilm formation and virulence in *S. aureus* is modulated by a toxin family of small amphipathic surfactant-like peptides known as the phenol-soluble modulins (PSMs; Peschel and Otto, 2013; Syed and Boles, 2014). The PSMs are highly versatile contributors to pathogenesis by, for example, lysing red and white blood cells; modulating responses of both innate and adaptive immunity; aiding in skin colonization and contributing to development of biofilm-associated infections (Peschel and Otto, 2013). To date, eight different PSMs have been characterized in *S. aureus* (four PSMα peptides, two PSMβ, δ-toxin, and PSM-mec) expression of which are positively regulated by the accessory gene regulator (AGR) quorum sensing system (Wang et al., 2007; Schwartz et al., 2014). The response regulator of AGR, AgrA, is central in PSM regulation. AgrA not only activates transcription of the AGR operon in an auto-feedback loop, but it also regulates expression of the AGR effector molecule RNAIII (that encodes for δ-toxin) as well as directly regulates *psm* genes by binding to their promoters (Queck et al., 2008; Cheung et al., 2014).

While the PSMα (α1–4) and δ-toxin are known to be the most cytolytic and immune modulating, they all play a role in the PSM-dependent spreading on epithelial surfaces (Tsompanidou et al., 2013) and also have been related to structuring and detachment of biofilms (Periasamy et al., 2012). Phenol-soluble modulins exist in a monomeric state, where their surfactant and lytic properties are more pronounced, and in an oligomerized, aggregated, and even fibrillated state, where they fold into amyloid-like structures. These PSM amyloid-like fibrils have been described to be non-cytolytic and speculated to contribute to biofilm robustness by providing biofilm resistance toward enzymatic degradation (Schwartz et al., 2012; Syed and Boles, 2014). Whether or not these PSM fibrils truly have a biological role in biofilm integrity and structuring remains unclear, though, as fibrillation has been difficult to demonstrate in bacterial cultures as other molecules, such as DNA, interfere with staining and potentially obscure their detection (Zheng et al., 2018). Furthermore, this non-specific attachment of PSMs to DNA may also explain biofilm resistance to enzymatic degradation (Zheng et al., 2018). Thus, the true biological impact of PSM fibrillation still remains unknown.

More recently, a new characteristic has been observed for the PSMs, namely their ability to reduce *S. aureus* persister cells (Xu et al., 2017; Bojer et al., 2018). Persister cells are subpopulations of cells that, in the absence of mutations, are highly tolerant to antibiotics at concentrations often 100 times greater than the minimum inhibitory concentration (MIC; Fisher et al., 2017; Balaban et al., 2019). These cells are generally thought to be in a state of dormancy (Song and Wood, 2020) and upon the removal of the antibiotic pressure will revert back to an antibiotic sensitive state. Persister cells may be generated spontaneously or due to environmental stress, such as starvation or drug exposure, and they are formed in both exponential and stationary phase (Balaban et al., 2019). Accordingly, persister cell formation is influenced by growth phase with higher frequencies being observed upon entering stationary phase (Lechner et al., 2012).

Bojer et al. (2018) showed that while the reduction in persister cell formation was dependent on PSMα, the synthetic PSMα3 alone had no effect on the formation of *S. aureus* persister cells. Furthermore, when PSMs were supplied as part of a spent supernatant from a bacterial culture, the active component reducing persister formation was in the fraction of molecules greater than 30 kDa, hinting to possible aggregation of the PSMs (Bojer et al., 2018). Thus, the questions as to which of the other alpha-PSMs are responsible for persister reduction and whether aggregation or fibrillation of PSMs could be driving this phenomenon still remain unanswered. In this study, we aim to address these questions by testing the modulating ability of individual or combined synthetic PSMα peptides and their equivalent amyloid-like fibrils on ciprofloxacin-selected *S. aureus* persister formation. Ultimately, this study will aid in obtaining a better understanding of this newly identified anti-persister role of PSM peptides in Staphylococcal biology, knowledge of which can lead to new avenues for the fight against chronic and persistent *S. aureus* infections.

## Materials and Methods

### Bacterial Strains, Growth Conditions, and Chemicals

The strains used in this study were *S. aureus* Newman WT (Laboratory strain collection), Newman Δ*agr* (Paulander et al., 2013), and Newman Δ*sucA* (Wang et al., 2018). Strains were taken from frozen stocks (−80°C) and grown on tryptic soy agar (TSA, Oxoid) at 37°C. Single colonies were then selected and grown as liquid cultures in tryptic soy broth (TSB, Oxoid) at 37°C while shaking at 200 rpm. Ciprofloxacin was supplied by Sigma-Aldrich (MO, USA), nisin by Sigma-Aldrich (N5764), and the PSMα peptides (N-formylated, >85% purity) were supplied by Royobiotech (Shanghai, China) with the sequences: PSMα1: fMet-MGIIAGIIKVIKSLIEQFTGK; PSMα2: fMet-MGIIAGIIKFIKGLIEKFTGK; PSMα3: fMet-MEFVAKLFKFFKDLLGKFLGNN, and PSMα4: fMet-MAIVGTIIKIIKAIIDIFAK.

### Phenol-Soluble Modulin Peptide Fibrillation

The PSM peptides were first dissolved in DMSO (final concentration 20 mg/ml) and then incubated at 2 mg/ml concentration in tris 10 mM, pH 7.5 in 96-well plate (Nunc, Thermo Fisher Scientific, Roskilde, Denmark) at 37°C in a Genios Pro fluorescence plate reader (Tecan, Männedorf, Switzerland). A clear crystal sealing tape (Hampton Research, Aliso Viejo, CA, USA) used to prevent solvent evaporation. The fibrillation process monitored by measuring fluorescent emission from thioflavin-T (ThT; 40 μM) as a well-known amyloid binding dye using excitation and emission at 448 and 485 nm, respectively. ThT-emission recorded every 5 min with 10 s orbital shake (300 rpm) before each cycle. All PSM-peptides show fibrillation except PSMα2 that could not form any fibrils (Supplementary Figure 1). For the incubation of PSMs together, 10 mg of each peptide was dissolved in DMSO (20 mg/ml for each), mixed, and then diluted in buffer (tris 10 mM) to reach 1 mg/ml of each peptide. The samples were incubated under same condition as described above.

### Persister Assays

Stationary phase persisters were determined as previously described (Wang et al., 2018) and with minor modifications, single colonies were inoculated into 2 ml TSB medium and incubated while shaking (200 rpm) at 37°C for 24 h in 15-ml centrifuge tubes. One milliliter of this 24 h culture was withdrawn and placed in a new 15-ml centrifuge tube, spun down, the supernatant discarded, and the pellet re-suspended in fresh TSB medium. At this point a time zero hours (T0) sample was taken for CFU quantification. The cells were then challenged with 40 μg/ml PSMα peptides and 100x MIC of ciprofloxacin for a further 24 h (T24) while shaking at 37°C. Hereafter, the tubes were centrifuged (12,000 *g* for 5 min), the pellet washed with 0.9% NaCl to remove any residual antibiotic, and subsequent known dilutions of the samples were plated on TSA plates and incubated for a further 24 h. The persister cell frequency was provided by determining the ratio between the CFU/ml count at T24 and T0. Exponential phase persisters were determined as described (Bojer et al., 2018) and with minor modifications, single colonies were inoculated into 2 ml TSB medium and incubated while shaking (200 rpm) at 37°C for 24 h in 15-ml centrifuge tubes. A sub-culture was then performed by diluting 1/1,000 in 2 ml fresh TSB and the cells were allowed to grow for 2.5 h to reach exponential growth. At this point T0, samples were taken for CFU quantification, after which the cells were exposed to 40 μg/ml PSMα peptides and 20x MIC ciprofloxacin and incubated for a further 24 h (T24) while shaking at 37°C. One milliliter of this T24 culture was withdrawn, washed with 0.9% NaCl, known dilutions were plated on TSA, and incubated at 37°C for 24 h. Persister frequency was calculated as for stationary persisters above.

Biofilm related persister cells were determined as described (Conlon et al., 2013), with minor modifications. Single colonies were inoculated into 2 ml TSB medium and incubated while shaking (200 rpm) at 37°C for 24 h in 15-ml centrifuge tubes. The cultures were diluted 1/1,000 in fresh TSB, the PSMα peptides added to a final concentration of 40 μg/ml, and 200 μl were distributed per well in a tissue culture treated polystyrene 96-well plate, and incubated at 37°C, static, for 24 h. The plate was split for T0 sampling and T24 sampling. The next day, for the wells destined for CFU quantification of T0, the medium was gently removed and the biofilms were washed twice with 100 μl of 0.9% NaCl, the biofilm was harvested, pooled per condition, and sonicated at 15 pulses, 500 ms, and 50% power using a Bandelin sonopuls HD2070/UW2070 (Bandelin electronics, Germany) apparatus. Known dilutions were plated on TSA and incubated at 37°C for 24 h. For the T24 sample wells, the medium was gently removed and fresh medium was added containing 100x MIC ciprofloxacin. The plate was incubated for further 24 h, static, at 37°C. CFU quantification was performed as for T0 samples. Persister frequency was calculated as described above.

A minimum of three biological replicates (overnight cultures originating from individual colonies) were included for each experiment.

### Membrane Potential Assay

Membrane potential was assessed by flow cytometry using the *Bac*light Bacterial Membrane Potential Kit (Invitrogen) as previously described (Vestergaard et al., 2018; Wang et al., 2018) with minor modifications. Overnight cultures grown in the presence of 40 μg/ml PSMα peptides were adjusted to an OD_600_ of 0.2. Then, 20 μl of the cultures were added to 1 ml of sterile filtered phosphate-buffered saline (PBS) and 10 μl of the fluorescent membrane potential indicator dye 3,3'-diethyloxacarbocyanine iodide [DiOC_2_(3)] was added to each tube. Samples were measured after 5 and 30 min of staining with the dye. Fluorescence was recorded using the BD Biosciences Accuri C6 flow cytometer (BD Biosciences, USA) counting 50,000 cells at a FSC threshold of 15,000 and at medium flow rate. After gating the stained cell populations, the ratio between red fluorescence (FL3 channel) and green fluorescence (FL1 channel) was calculated as an indicator of the membrane potential. The assay was verified using the protonophore carbonyl cyanide *m*-chlorophenyl hydrazine (CCCP) at a concentration of 5 μM. At least three biological replicates were assayed per condition.

### Propidium Iodide Staining for Leaky/Damaged Cells

Leaky or damaged cells were assessed by flow cytometry using Propidium Iodide Ready Flow Reagent as per manufacturer’s instructions (Invitrogen) with minor alterations. Samples were prepared as for membrane potential assay in sterile PBS and two drops of the PI Ready Flow reagent were added per sample and left to incubate at room temperature for 15 min. Red Fluorescence (FL3 channel) was recorded using the BD Biosciences Accuri C6 flow cytometer (BD Biosciences, USA). At least three biological replicates were assayed per condition.

### Statistical Analysis

Significant differences were calculated by two-tailed Student’s *t*-test. Statistical analysis was performed using GraphPad Prism 7 (GraphPad Software, Inc., La Jolla CA, USA). Persister frequencies were log transformed prior to statistical analysis to normalize the variance. Values of *p* < 0.05 were considered statistically significant.

## Results

### PSMα Peptides Reduce Persister Cell Frequencies in Planktonic and Biofilm Grown *Staphylococcus aureus*

**Figure 1:**
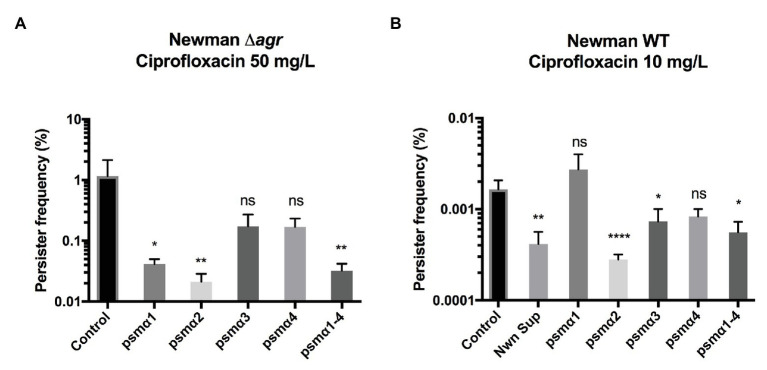
Phenol-soluble modulins reduce *Staphylococcus aureus* persisters. Strain Newman Δ*agr* stationary phase cultures **(A)** or wild type (WT) exponential phase cultures **(B)** were exposed to 40 μg/ml of synthetic PSMα 1–4 peptides. Persisters were selected for using 100x MIC ciprofloxacin for stationary and 20x MIC for exponential cultures. All treatments were compared to the untreated control and 25% WT supernatant (containing endogenously produced PSMs) was added as a positive control for the exponential phase cultures. All data represent the mean persister cell frequencies ±SD of nine biological replicates. (^*^ *p* < 0.05; ^**^ *p* < 0.01; ^****^ *p* < 0.0001).

To examine the individual contribution of the PSM peptides to persister cell formation, we examined the PSMα peptides, either alone or in combination and at the physiologically relevant concentration of 40 μg/ml (Hongo et al., 2009; Ebner et al., 2017). Persister cell formation was monitored upon exposure to lethal doses of ciprofloxacin, where we previously have seen persister cells forming (Wang et al., 2018). As we wanted to investigate the effect of externally added PSM peptides on persister frequency, we employed a Newman Δ*agr* mutant that does not produce endogenous PSMs (Queck et al., 2008). Our result shows that PSMα2 was the most effective at reducing Newman persister cell frequency in stationary phase upon ciprofloxacin selection (Figure 1A). PSMα2 at a concentration of 50 μg/ml, and to a lesser extent PSMα3 and α4, have previously been reported to induce non-classical protein excretion through cytoplasmic leakage by damaging the cell membrane in exponential phase cells of the *S. aureus* strain USA300 (Ebner et al., 2017). To rule out that the reduction in persister frequency observed was an artifact of any adverse effects on cell viability due to the cell membrane damage, the growth and CFU of cultures treated with PSMα peptides at the tested concentration of 40 μg/ml (Supplementary Figures 2A,B) as well as propidium iodide (PI) staining analyzed by flow cytometry to measure cell leakage (Supplementary Figure 2C) were evaluated. No effect on growth or CFU count was observed, neither was any significant cell damage, indicating that the reduction of the persister cell frequency observed was not due to adverse effects of PSMα2-induced cell damage. Apart from PSMα2, PSMα1 and the mixture of all four PSMα peptides (PSMα1–4) also contributed to a significant decrease in persister cell frequency.

Persister cells have also been observed to appear stochastically during exponential growth (Balaban et al., 2019). Here, we sought to determine whether the PSMα peptides could influence persister development in this growth phase. Once again PSMα2 had a significant impact in reducing the frequency of persister development (Figure 1B), followed by the mixture of all four PSMα peptides (PSMα1–4).

**Figure 2:**
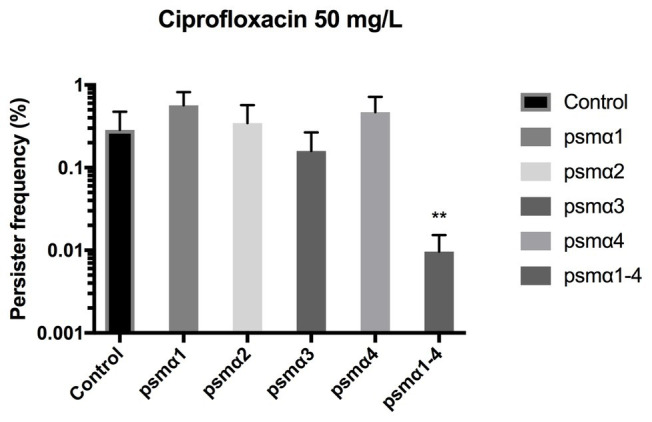
Combined PSMα1–4 reduces *Staphylococcus aureus* persister development in biofilms. Strain Newman Δ*agr* static 96-well biofilms were grown in the presence of 40 μg/ml of synthetic PSMα 1–4 peptides for 24 h. Persisters were selected for using 100x MIC ciprofloxacin. All treatments were compared to the untreated control. All data represent the mean persister cell frequencies ±SD of three biological replicates. (^**^ *p* < 0.01).

*Staphylococcus aureus* is efficient in colonizing tissues and abiotic surfaces in a biofilm state (Boles and Horswill, 2011). With this in mind, we were interested to see whether the PSMα peptides might also reduce persister cell populations in biofilms. Once again, to eliminate the influence of endogenously produced PSMs, Newman Δ*agr* was used and the PSMα peptides were added exogenously. When we monitored the impact of PSMs on biofilm in a previously established biofilm system (Conlon et al., 2013), we observed that it was only in the presence of all four alpha PSMs that a significant reduction in biofilm-related persister cell frequencies occurred upon ciprofloxacin challenge (Figure 2). Thus, depending on growth phase, the peptides are active in reducing persister cells to a variable degree and the combination reduces biofilm related persisters.

### PSMα Peptide Fibrils Do Not Reduce *Staphylococcus aureus* Persisters

**Figure 3:**
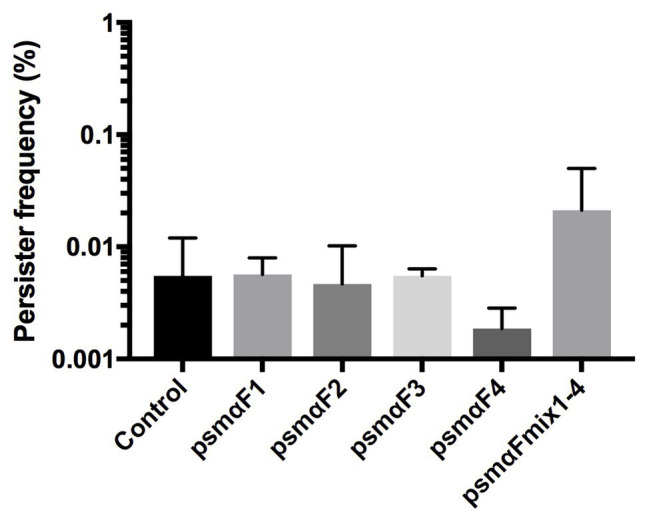
*Staphylococcus aureus* persister cell frequency is not affected by fibrillated (F) PSMα1–4 peptides. Synthetic PSMα 1–4 peptides were allowed to fibrillate in milliQ water for 48 h either individually or as a combination of all four peptides. Strain Newman stationary phase cultures were subjected to fibrillated (F) PSMα1–4 peptides at a concentration of 40 μg/ml for 24 h. Persisters were selected for using 100x MIC ciprofloxacin. All treatments were compared to the untreated control. All data represent the mean persister cell frequencies ±SD of nine biological replicates.

In the recent study by Bojer et al. (2018), where PSMs were identified as being able to reduce the number of persister cells, the activity was observed in the fraction of molecules greater than 30 kDa, hinting to the possibility that the PSMs were present in an aggregated or fibrillated form (Bojer et al., 2018). Therefore, we examined if it is the monomer or the fibrillated form of PSMα peptides (see Supplementary Figure 1) that affects persister cell formation. Our results revealed that there was no reduction in persister frequencies with the fibrillated PSMα peptides compared to the untreated control (Figure 3). Thus, we conclude that the PSMα peptide fibrils are not able to modulate persister cell formation.

### PSMα Peptides Reduce Persister Cells in a High Persister Forming Mutant

**Figure 4:**
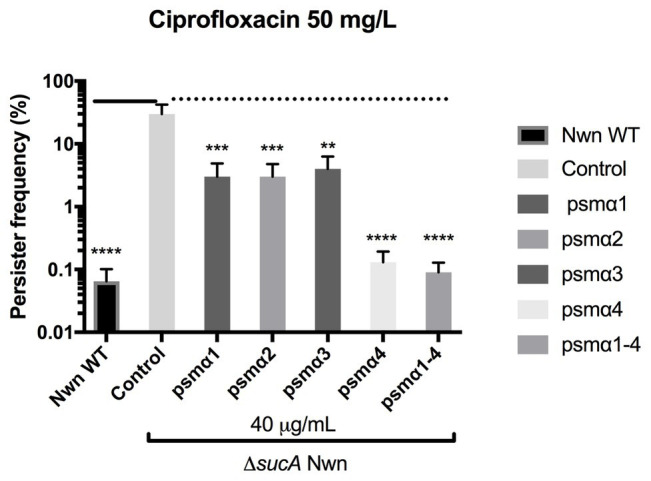
*Staphylococcus aureus* PSMs reduce persister cell formation in a high persister mutant strain. Stationary cultures of strain Newman WT (Nwn) or a Δ*sucA* mutant derivative were exposed to 40 μg/ml of synthetic PSMα 1–4 peptides. Persisters were selected for using 100x MIC ciprofloxacin. All treatments were compared to the untreated control. All data represent the mean persister cell frequencies ±SD of nine biological replicates. (^**^ *p* < 0.01; ^***^ *p* < 0.001; ^****^ *p* < 0.0001).

Multiple genes and processes are involved in persister cell formation and in *S. aureus*, it has been reported that mutations in key enzymes involved in the Krebs cycle result in high persister cell frequencies (Wang et al., 2018). These mutants were found to have reduced membrane potential, which was suggested to contribute to the persister formation (Wang et al., 2018). To address the mechanism of action employed by the PSMα peptides in reducing persister frequencies, we looked at their effect on persister formation in a *S. aureus* Newman strain harboring an insertion mutation in the *sucA* gene encoding for a subunit of the α-ketoglutarate dehydrogenase enzyme of the Krebs cycle (Tretter and Adam-Vizi, 2005). In accordance with reported data, the *sucA* mutant formed three log-folds more persister cells over the WT when challenged with 100x MIC ciprofloxacin (Figure 4). Furthermore, when in the presence of the PSMα peptides there was a 10-fold decrease in persisters for α1, α2, and α3, and a 300-fold decrease for α4 and the combination of α1–4 peptides. These data show that persister cells formed in a Krebs cycle mutant are still susceptible to PSMα peptides and that all PSMα peptides are capable of reducing persister cell formation.

Reduced membrane potential is a common mechanism associated with increased drug resistance due to decreased uptake of an antibiotic (Reygaert, 2018; Vestergaard et al., 2018). Therefore, we hypothesized that perhaps the PSMs were increasing the membrane potential of the cells, thus making them more susceptible to ciprofloxacin and inducing cell death rather than tolerance. To this end, membrane potential of both the WT and the *sucA* mutant in the presence of the PSMα peptides was monitored using the fluorescent probe DiOC_2_(3) and flow cytometry, while simultaneous sampling was performed to monitor the persister frequencies in these samples. Here, we were unable to detect any effect of PSMs on membrane potential despite affecting persister formation (data not shown). Thus, the mechanism by which the PSMα peptides reduce persister cell development still remains elusive.

## Discussion

Persister cells are a subpopulation of cells that in a state of dormancy have been characterized by low ATP levels (Conlon et al., 2016), reduced membrane potential (Wang et al., 2018), and can be triggered by, for example, toxins (Wilmaerts et al., 2018) or ribosome inactivation (Song and Wood, 2020). We previously demonstrated that persister cells are susceptible to PSMα toxins (Bojer et al., 2018) and, here, this observation has been investigated further. Importantly, we find that individually, all four alpha peptides have activity against persister cells when examined in a mutant that forms high levels of persister cells due to a mutation in the Krebs cycle, with PSMα4 being particularly potent. When monitored in planktonic, WT cells, the PSMα2 most consistently showed the greatest activity. Currently, it is unclear why the activities of the PSMα peptides differ between mutant and WT cells but the greater hydrophobicity of PSMα4 (as assessed by https://www.peptide2.com/N_peptide_hydrophobicity_hydrophilicity.php) compared to the other peptides may enhance the activity of this particular PSM toward mutant cells, where the membrane potential is reduced by the *sucA* mutation. In biofilms, it was only the combination of all four PSMα peptides that showed an effect and this combination also showed strong persister reducing activity when planktonic cells were assessed. Initially, we speculated that the efficacy of the peptide combination may be related to fibrillation as Zheng et al. observed greater degree of fibrillation in mixtures of peptides (Zheng et al., 2018). However, in our hands, we saw most pronounced fibrillation by PSMα3 although this peptide displayed least activity in the persister assays and vice versa for PSMα2 (Supplementary Figure 1). Further, all fibrillated PSMs lost their activity against persister cells. Thus, if fibrillated forms of PSMs occur in bacterial cultures, they may serve as a reservoir of PSMs in a state that is inactive against persister cells but could be mobilized for future use. Such storage has been proposed for peptide hormones in an amyloid state (Maji et al., 2009) that are released by a change in pH (Nespovitaya et al., 2016), and it has been suggested for PSMs in relation to their cytotoxic activity against host neutrophils (Wang et al., 2007; Laabei et al., 2014).

PSM peptides have previously been demonstrated to have antimicrobial activity. For example, proteolytically processed PSMα1 and PSMα2 exhibited considerable activity against *Streptococcus pyogenes* (Joo et al., 2011). They also appear to have lytic activity against *S. aureus* itself as they were found to be responsible for the “non-classical” excretion of proteins by which cytoplasmic proteins are released following membrane damage inflicted particularly by PSMα2 (Ebner et al., 2017). In our assays, we did not observe any reduction in viability of the bacterial cultures upon treatment with PSMα peptides, but we speculate that the persister state may be associated with changes in the bacterial membrane that increase the susceptibility to PSMα mediated disruption. If an altered membrane structure or composition is associated with persister cells, it will be more prevalent in cells with decreased membrane potential (Wang et al., 2018) and, particularly, those cells will be more susceptible to PSMα4 compared to the other peptides. Increased understanding of what such membrane changes may involve may provide us with a window of opportunity to target persister formation and render bacterial pathogens more susceptible to antimicrobial chemotherapy.

## Data Availability Statement

The raw data supporting the conclusions of this article will be made available by the authors, without undue reservation.

## Author Contributions

MB, MSB, and HI designed the study and wrote the manuscript. MB, MSB, and ZN conducted the experimental work. MB, MSB, ZN, MV, RM, DO, and HI analyzed the data. All authors contributed to the article and approved the submitted version.

### Conflict of Interest

The authors declare that the research was conducted in the absence of any commercial or financial relationships that could be construed as a potential conflict of interest.
