# Supplementary material for: Phenol-Soluble Modulins Modulate Persister Cell Formation in Staphylococcus aureus
Source: Front Microbiol. 2020 Nov 9;11:573253. doi: 10.3389/fmicb.2020.573253 (PMC7680730; doi:10.3389/fmicb.2020.573253)

Supplementary Material

Supplementary Figure 1.

Fibrillation of PSM peptides monitored by using ThT-fluorescence

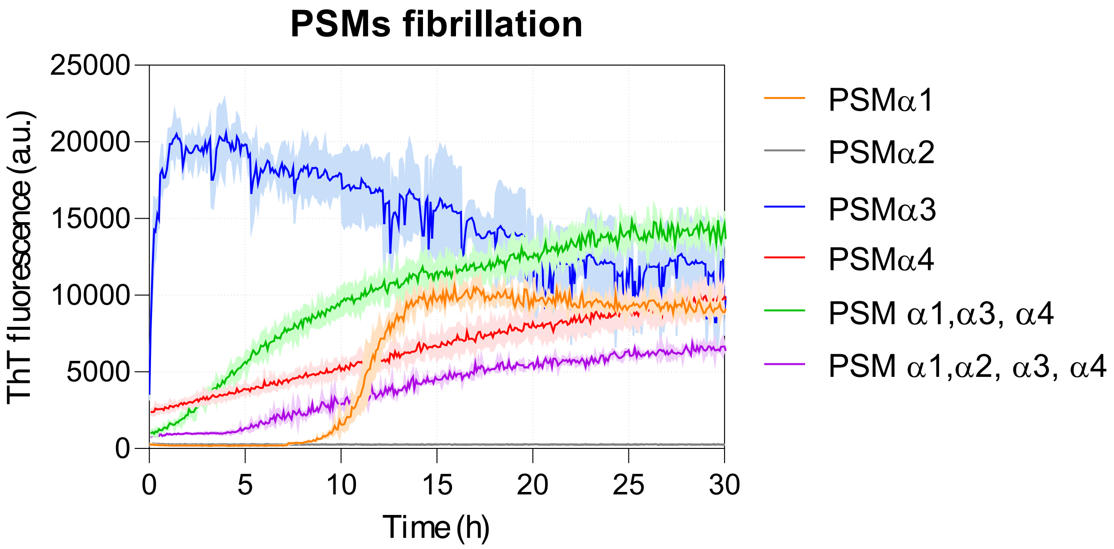

Supplementary Figure 2

Synthetic *S. aureus* Phenol Soluble Modulins are not toxic to *S. aureus* strain Newman at the tested concentration of 40 µg/mL. (**a**) Growth of *S. aureus* strain Newman in the presence of PSMα1-4 synthetic peptides as measured over 24 hours and assessed by OD_{600}. (**b**) CFU/mL counts of stationary, exponential and biofilm samples exposed to PSMα1-4 synthetic peptides prior to persister cell selection with Ciprofloxacin treatment. (**c**) Percentage of dead cells after treatment of synthetic PSMα1-4 peptides as assessed by staining of damaged/leaky cell membranes by propidium iodide. Nisin and DMSO were included as positive and vehicle controls respectively. All data represent the mean persister cell frequencies ±SD of 3 biological replicates.

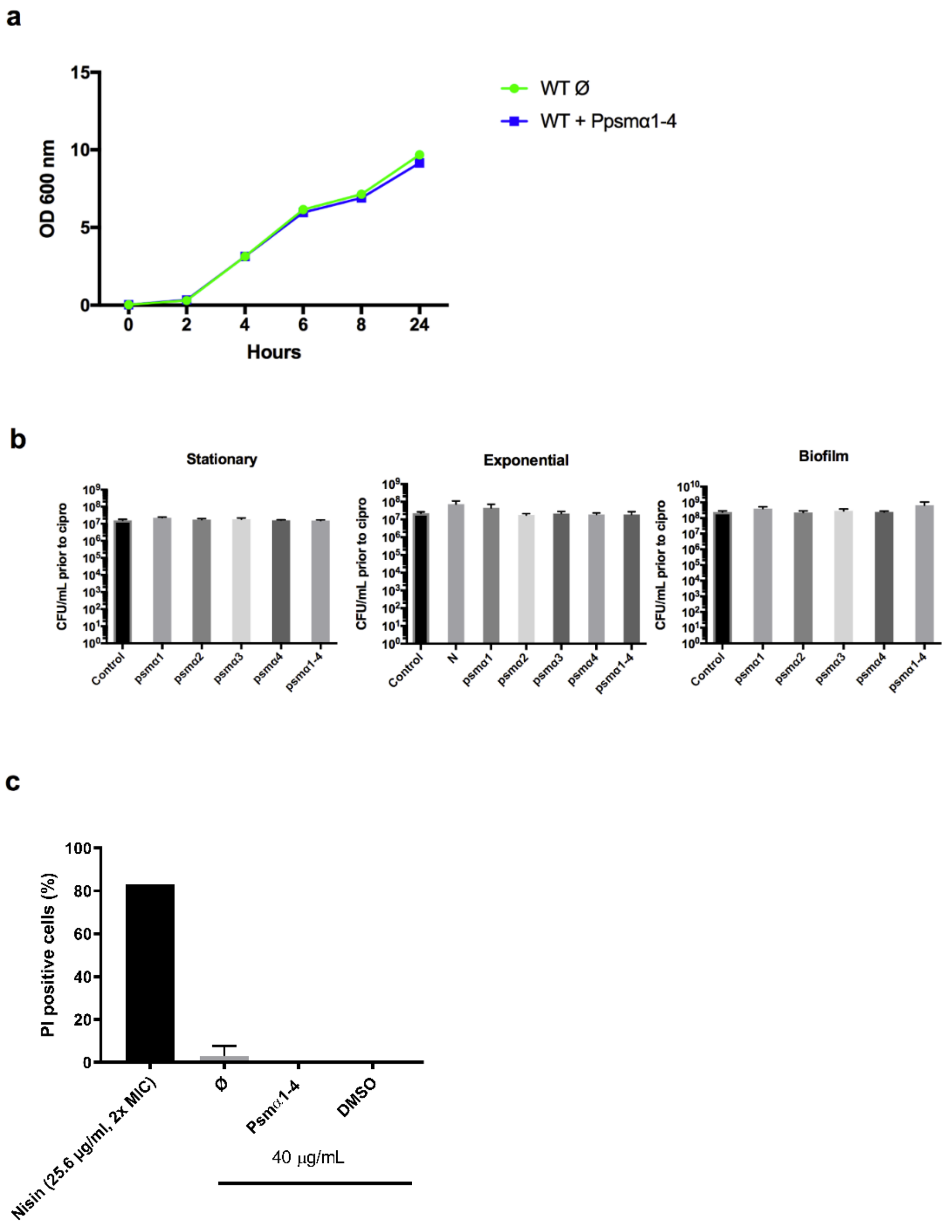

Supplement: Supplementary file 1 [file Data_Sheet_1.pdf]
